# Cardiomyocyte-Specific RIP2 Overexpression Exacerbated Pathologic Remodeling and Contributed to Spontaneous Cardiac Hypertrophy

**DOI:** 10.3389/fcell.2021.688238

**Published:** 2021-10-18

**Authors:** Jing-jing Yang, Nan Zhang, Zi-ying Zhou, Jian Ni, Hong Feng, Wen-jing Li, Shan-qi Mou, Hai-ming Wu, Wei Deng, Hai-han Liao, Qi-zhu Tang

**Affiliations:** ^1^Department of Cardiology, Renmin Hospital of Wuhan University, Wuhan, China; ^2^Hubei Key Laboratory of Metabolic and Chronic Diseases, Wuhan, China; ^3^Department of Geriatrics, Renmin Hospital of Wuhan University, Wuhan, China

**Keywords:** cardiac remodeling, spontaneous cardiac remodeling, RIP2, TAK1, MAPK

## Abstract

This study aimed to investigate the role and mechanisms of Receptor interacting protein kinase 2 (RIP2) in pressure overload-induced cardiac remodeling. Human failing or healthy donor hearts were collected for detecting RIP2 expression. RIP2 cardiomyocyte-specific overexpression, RIP2 global knockout, or wild-type mice were subjected to sham or aortic banding (AB) surgery to establish pressure overload-induced cardiac remodeling *in vivo*. Phenylephrine (PE)-treated neonatal rat cardiomyocytes (NRCMs) were used for further investigation *in vitro*. The expression of RIP2 was significantly upregulated in failing human heart, mouse remodeling heart, and Ang II-treated NRCMs. RIP2 overexpression obviously aggravated pressure overload-induced cardiac remodeling. Mechanistically, RIP2 overexpression significantly increased the phosphorylation of TAK1, P38, and JNK1/2 and enhanced IκBα/p65 signaling pathway. Inhibiting TAK1 activity by specific inhibitor completely prevented cardiac remodeling induced by RIP2 overexpression. This study further confirmed that RIP2 overexpression in NRCM could exacerbate PE-induced NRCM hypertrophy and TAK1 silence by specific siRNA could completely rescue RIP2 overexpression-mediated cardiomyocyte hypertrophy. Moreover, this study showed that RIP2 could bind to TAK1 in HEK293 cells, and PE could promote their interaction in NRCM. Surprisingly, we found that RIP2 overexpression caused spontaneous cardiac remodeling at the age of 12 and 18 months, which confirmed the powerful deterioration of RIP2 overexpression. Finally, we indicated that RIP2 global knockout attenuated pressure overload-induced cardiac remodeling *via* reducing TAK1/JNK1/2/P38 and IκBα/p65 signaling pathways. Taken together, RIP2-mediated activation of TAK1/P38/JNK1/2 and IκBα/p65 signaling pathways played a pivotal role in pressure overload-induced cardiac remodeling and spontaneous cardiac remodeling induced by RIP2 overexpression, and RIP2 inhibition might be a potential strategy for preventing cardiac remodeling.

## Introduction

Pathological cardiac remodeling is a common pathological process for all cardiovascular diseases to develop and progress into heart failure (Wu et al., 2017; Morita and Komuro, 2018). Despite advances in medical technology and medicine discovery, heart failure remains to be a severe condition with high morbidity and mortality worldwide and a satisfactory treatment strategy is still to be expected (Yancy et al., 2017). Studies have suggested that targeting cardiac remodeling at an early stage might be a prospective strategy for preventing the development and progress of heart failure (Wu et al., 2017; Gibb and Hill, 2018; Gonzalez et al., 2018). Although cardiac remodeling has drawn much attention, the underlying mechanisms remain to be largely unknown. More studies are needed for clarifying underlying mechanisms in cardiac remodeling.

Receptor interacting protein kinase 2 (RIP2) belongs to Tyrosine kinase-like family (Humphries et al., 2015). RIP2 contains a homologous kinase domain in its N-terminal, a caspase activation and recruitment domain in its C-terminal and a bridging intermediate domain between N- and C- terminal (Humphries et al., 2015). RIP2 could receive signal transduction from nucleotide-binding oligomerization domain-containing protein 2 (NOD2) (Humphries et al., 2015). Muramyl dipeptide (MDP) combined to the leucine-rich repeat regions of NOD2 and mediated NOD oligomerization (Humphries et al., 2015). Oligomerized NOD proteins bound to the CARD domains of RIP2 kinase, which resulted in RIP2 polyubiquitination and led to recruitment of TAK1 and IKK complexes to activate IKK/NF-κB and MAPKs/AP1 signaling pathways (Humphries et al., 2015). Both IKK/NF-κB and MAPKs/AP1 are key signaling pathways involved in pathological remodeling (Rose et al., 2010; Wu et al., 2017); however, it remains to be defined whether RIP2 overexpression could exacerbate pathological cardiac remodeling *via* these signaling pathways.

Several studies have explored the role and mechanisms of RIP2 in different cardiovascular diseases. Jacquet et al. (2008) demonstrated that RIP2 deficiency showed no obvious effects on ischemia-associated cardiac function and infarction size, because RIP2 was not the mediator of downstream signaling in ischemia-treated cardimyocyte (Jacquet et al., 2008). In another study on heart ischemia, investigators presented that RIP2 deficiency enhanced myocardial damage area and markedly decreased cardiac function *via* promoting vascular endothelial growth factor (VEGF) expression for activating ERK1/2 and resulting in endothelial permeability and inflammation (Andersson et al., 2015). However, Li et al. (2015) demonstrated that RIP2 deficiency significantly attenuated ventricular remodeling after ischemia *via* inhibiting the NF-κB and p38 MAPK signaling pathways. Recently, Zhao et al. (2017) demonstrated that global knockout of RIP2 attenuated pressure overload-induced cardiac remodeling *via* reducing toll-like receptor4/myeloid differentiation factor 88/nuclear factor kappa B (TLR4/MyD88/NF-K b) and MAPKs activity.

According to these descriptions, RIP2 was considered to play important roles in cardiovascular disease; however, the exact role and mechanisms of RIP2 in cardiomyocytes remained to be controversial. In this study, we presented that pro-hypertrophy stimuli promoted RIP2 overexpression *in vivo* and in *vitro*. With the pathological cardiac hypertrophy mouse model, we firstly demonstrated that cardiomyocyte-specific expression of RIP2 could exacerbate pressure overload-induced pathological cardiac hypertrophy. Moreover, RIP2 overexpression caused spontaneous cardiac hypertrophy and RIP2 knockout *in vivo* and silence *in vitro* could attenuate pathological cardiac remodeling and NRCM hypertrophy, respectively.

## Materials and Methods

### Animals

All experimental procedures in this study were approved by the Animal Care and Use Committee of Renmin Hospital of Wuhan University and were performed in accordance with the National Institutes of Health (NIH) Guide for the Care and Use of Laboratory Animals.

To generate cardiomyocyte-specific RIP2 transgenic mice, full-length RIP2 cDNA was cloned into plasmid under control of cardiac α-MHC promoter (Supplementary Figure 1A). Then, the plasmid was linearized and was microinjected into fertilized mouse embryos for giving birth to cardiomyocyte-specific RIP2 overexpression mice. These transgene mice were identified by PCR analysis of tail genomic DNA. Primers used for transgene mice identification in this manuscript were listed as follows: forward: ATCTCCCCCATAAGAGTTTGAGTC, and reverse: ATTGTCCCTCCTTCTGGTGCAG. According to the results of Western blots, the mice of RIP2 transgene (TG) line 2 were chosen for the following experiments (Supplementary Figure 1B). Non-transgenic mice (NTG) born in the same nest were used as control group. Global RIP2-knockout mice (007017) were purchased from the Jackson Laboratory and were mated with C57B/6L for nine generations. Male mice (age 8–10 weeks, weight 24.5–25.5 g) were used for subsequent experiments. All animals were raised in specific-pathogen-free (SPF) conditions in the Cardiovascular Research Institute of Wuhan University. Mice were allowed access to food and water freely with a 12-h light/dark cycle.

### Established Cardiac Hypertrophy Mouse Model

A cardiac hypertrophy mouse model was established by aortic banding (AB) surgery according to our previous publications (Liao et al., 2015; Yuan et al., 2016). Briefly, mice were anesthetized by intraperitoneal injection of pentobarbital sodium (50 mg/kg, Sigma). After adequate anesthesia without a toe pinch reflex, the left chest was opened at the second intercostal space *via* blunting dissection to find out the thoracic aorta. A 27-gauge needle was tied to the aorta with a 7-0 silk suture. After ligation of the aorta and needle, the needle was quickly removed and the aortic constriction was confirmed by Doppler analysis. Animals in the sham group were subjected to the same procedure without ligation. Surgery operation and analyses were carried out in a blinded manner. Angiotensin II (Ang II, 1.1 mg/kg/day) was infused with Alzet Osmotic Pumps 2002 to induce the Ang II-induced cardiac hypertrophy mouse model.

### Specific Inhibitor Administration

To clarify the signaling pathways located downstream of RIP2, a specific inhibitor for TAK1 (Takinib, 253863-19-3, Med ChemExpress, United States) was resolved in DMSO and then was administered to NTG and TG mice (5 mg/kg/day) for 4 weeks to inhibit TAK1 activity. The mice in the control group (VEH group) were treated with an identical dose of DMSO to the TAKi group (the mice treated with inhibitor of TAK1). The treatment was started after 3 days of AB surgery.

### Mouse Heart Harvest

Four weeks after surgery, mouse cardiac function was examined by echocardiography and pressure-volume loop under inhalation anesthesia with 1.5% isoflurane. Then, the mice were sacrificed by cervical dislocation. The body weight (BW), heart weight (HW), lung weight (LW), and tibia length (Tib) were recorded for the following analysis. Mouse hearts were randomly assigned into a pathological staining group or a molecular analysis group.

### Echocardiography for Cardiac Function Analysis

Echocardiography was carried out by a MyLab 30CV ultrasound (Biosound Esaote Inc.) equipped with a 10-MHz linear array ultrasound transducer. Echocardiography was performed under continuous anesthesia with 1.5% isoflurane. M-mode tracings were recorded at the mid-papillary muscle level at the short axis of the left ventricle (LV). The LV end-systolic diameter (LVEDs) and LV end-diastolic diameter (LVEDd) were measured by M-mode tracing with a sweep speed of 50 mm/s. LV ejection fraction (EF) and fractional shortening (FS) were calculated according to LVEDs and LVEDd.

### Pressure-Volume Loop for Hemodynamic Analysis

Hemodynamic changes were assessed in mice anesthetized by inhaling 1.5% isoflurane. A microtip catheter transducer (SPR-839, Millar Instruments, Houston, TX, United States) was inserted into the carotid artery until it arrived at the left ventricle. Heart rates and pressure signals were recorded *via* an ARIA pressure-volume conductance system coupled to a Powerlab/4SP A/D converter. The data collected from hemodynamics measurement were analysis by PVAN data analysis software.

### Histological Staining Analysis

Mouse hearts were harvested and were immersed into 10% KCl solution for arresting in its diastole phase. Then, the mouse hearts were fixed in 10% formalin for 12 h and were dehydrated according to standard histological protocols. Dehydrated hearts were embedded in paraffin for preparing 4- to 5-μm tissue slices. Hematoxylin–eosin (HE) staining was performed to observed heart cross-sectional area and calculate cardiomyocyte sizes. Picrosirius red (PSR) staining was performed to assess the fibrosis in mouse heart. HE and PSR staining images were obtained using the light microscopy (Nikon). The histological analysis was performed using a quantitative digital image analysis system (Image-Pro Plus, version 6.0).

### Cell Culture and Treatment

The neonatal rat cardiomyocytes (NRCMs) were isolated from 1- to 3-day-old Sprague–Dawley (SD) rats using the methods as described in previous studies (Liao et al., 2015; Yuan et al., 2016). Briefly, neonatal rat hearts were minced into 1- to 2-mm^3^ pieces and were digested with PBS containing 0.1% trypsin and 0.05% collagenase type II. Differential attachment culture was performed to separate cardiac fibroblasts from cardiomyocytes. NRCMs were counted and seeded at a density of 1 × 10^6^ cells per well in six-well plates or 2 × 10^5^ cells per well in 12-well plates. After 36 h culture in medium with BrdU, NRCMs were transfected with adenoviruses carrying a target gene sequence for another 24 h.

In this study, adenoviruses were constructed containing sequences encoding human RIP2 and short-hairpin RNA-targeting at RIP2 (Ad-shRNA RIP2). The sequences used for RIP2 knockdown were listed as follows: ShRNA1 (Top Strand: 5′-CACCGCTCGACAGTGAAAGAAATGACGAATCATTTCT TTCACTGTCGAGC-3′, Bottom Strand: 5′-AAAAGCTCGA CAGTGAAAGAAATGATTCGTCATTTCTTTCACTGTCGAG C-3′), ShRNA2 (Top Strand: 5′-CACCGCATAGTTACTGAA TACATGCCGAAGCATGTATTCAGTAACTATGC-3′, Bottom Strand: 5′-AAAAGCATAGTTACTGAATACATGCTTCGGCAT GTATTCAGTAACTATGC-3′), shRNA 3 (Top Strand: 5′-CACCGCATGATATATACAGCTATGCCGAAGCATAGCTGTA TATATCATGC-3′, Bottom Strand: 5′-AAAAGCATGATA TATACAGCTATGCTTCGGCATAGCTGTATATATCATGC-3′). Similar adenoviruses carrying sequences of green fluorescent protein and short hairpin RNA was used for control. NRCMs were transfected with adenoviruses with a multiplicity of infection (MOI) of 50 per cell for 4 h and then the culture medium containing adenoviruses was removed and fresh complete culture medium was added for another 24 h of culturing. Then, NRCMs were treated with angiotensin II (Ang II, 1 μM) or phenylephrine (PE, 50 μM) for succeeding experiments.

### Immunofluorescence Staining

Immunofluorescence staining was performed to examine the specific overexpression of RIP2 in cardiomyocytes of mouse hearts. Briefly, frozen heart sections were fixed for 15 min at room temperature after covering sections with 4% formaldehyde. After rinsing three times for 5 min for these fixed sections, blocking buffer was added for 60 min. Then, these sections were incubated with primary antibodies (anti-RIP2, 1:200 and anti-α-actin, 1:200) overnight at 4°C. The incubated sections were rinsed three times for 5 min the next day and then were incubated with fluorochrome-conjugated secondary antibody (1:1,000) for 1 h at room temperature in the dark. After rinsing three times for 5 min in PBS, these sections were covered with coverslip slides with Prolong Gold Antifade Reagent for taking pictures with fluorescence microscope in the dark.

Immunofluorescence staining was also performed to assess cardiomyocyte hypertrophy *in vitro*. Briefly, NRCMs were transfected with adenoviruses or siRNA for 24 h and then were treated with PE for another 48 h. The cells were washed with PBS, fixed with 4% paraformaldehyde for 15 min, and then permeabilized with 0.2% Triton X-100 (Amresco, 0649) for 5 min. Cells were blocked in 10% goat serum and then stained with α-actinin (Millipore, 05-384, 1:100) for overnight at 4°C. After discarding the primary antibody the next day, cells were incubated with secondary antibody conjugated with Alexa fluor 488 (1:200) for 1 h. After discarding the secondary antibody, cells were covered by glass slides with DAPI (Invitrogen, S36939) for fluorescent photography in dark. The surface area of cardiomyocytes was assessed using a quantitative digital image analysis system (Image Pro-Plus, version 6.0).

### Co-immunoprecipitation

To test the interaction between TAK1 and RIP2 in HEK 293 cells, human TAK1 and RIP2 gene was cloned into pcDNA5-HA-C1 and pcDNA5-Flag-C1, respectively. pcDNA5-TAK1-HA (2 μg) and pcDNA5-RIP2-Flag (2 μg) was co-transfected into HEK293 cells. After 24 h of expression, cells were harvested and lysed in lysis buffer containing protease inhibitor.

To test PE promoting the interaction of RIP2 and TAK1, NRCMs were transfected with AdRIP2 with 50 MOI for 4 h and then the culture medium containing adenoviruses was removed and fresh complete culture medium was added for another 24 h of culturing. Then, NRCMs were treated with PE (50 μM) for 24 h. Cells were harvested and lysed in lysis buffer containing protease inhibitor.

To clear non-specific protein binding, about 1 mg of protein of each sample was incubated with normal rabbit immunoglobulin G (1 μg) and protein A/G-agarose beads (20 μl) (Thermo Fisher Scientific, United States) for 1 h at 4°C, and then supernatants were collected after 14,000 *g* centrifugation for 5 min. Collected supernatants were incubated with the indicated primary antibody (1 μg) overnight at 4°C. The next day, 40 μl of protein A/G-agarose beads was added for another 3 h with gentle shaking. After 1000 *g* centrifugation for 5 min, protein A/G-agarose beads were collected and boiled in loading buffer for the next Western blots analysis.

### Western Blot Analysis

Tissue or cell proteins were prepared according to published protocols (Liao et al., 2015; Yuan et al., 2016). Briefly, mouse heart tissue or NRCMs were lysed in RIPA lysis buffer for total protein extraction. BCA Protein Assay Kit was used for quantifying protein concentration. Proteins (50 μg/sample) from different samples were used for SDS-PAGE electrophoresis and subsequently transferred to PVDF membrane (Millipore). After blocking with 5% BSA for 1 h, blots were then incubated with primary antibodies overnight at 4°C. The next day, these blots were incubated with secondary antibody conjugated with peroxidase (1:10,000, the Jackson Laboratory) for 1 h at room temperature. All blots were visualized using ChemiDocTM XRS+ (Bio-Rad). The bands were quantified and analyzed using Image-Pro Plus 6.0. The expression of specific proteins was normalized to corresponding GAPDH before relative quantitative analysis. The primary antibodies used in this study were purchased from Cell Signaling Technology (CST): GAPDH (#2118, 1:1,000), p-ERK1/2^*Thr202/Tyr204*^ (#4370, 1:1,000), T-ERK1/2 (# 4695, 1:1,000), p-p38^*Thr180/Tyr182*^ (#4511, 1:1,000), T-p38 (#9212, 1:1,000), p-JNK^*Thr*183/*Tyr*185^ (#4668, 1:500), T-JNK (#9258, 1:1,000), RIP2 (#4142, 1:1,000), p-TAK1^*Thr184/187*^ (#4508, 1:500), T-TAK1 (#5206, 1:500), TGF-β, p-smad3^*ser423/425*^ (#8769, 1:500), and T-smad3 (#9513, 1:1,000).

### Real Time Quantitative Polymerase Chain Reaction

Total RNA was extracted from the left mouse ventricular cells using TRIzol reagent (Invitrogen, United States) and was converted into cDNA using the Transcriptor First Strand cDNA Synthesis Kit (Roche, 04896866001) according to the manufacturer’s protocol. SYBR Green (Roche, 04707516001) was used to detect the amplification of target genes. *Gapdh* gene expression was used as internal reference gene. The sequences of primers for real time quantitative polymerase chain reaction (RT-PCR) were provided in our previously published article (Liao et al., 2015).

### Human Heart Samples

Human failing hearts were obtained from the left ventricular wall of dilated cardiomyopathy patients accepting heart transplants. Normal heart samples were collected from the left ventricular wall of healthy donor hearts without a successful matched patient on time. All procedures associated with use of human heart samples were approved by Renmin Hospital of Wuhan University, Wuhan, China. Procedures complied strictly with the principles outlined in the Declaration of Helsinki. Informed consent was signed by patients or families of prospective heart donors.

### Statistical Analysis

Data are presented as mean ± SEM. Statistical comparisons among different groups were performed by one-way analysis of variance (ANOVA) followed by a Tukey’s *post hoc* test. The differences between two groups are determined by ANOVA followed by a two-tailed unpaired Student’s *t*-test. All data were analyzed using SPSS 22.0 software (SPSS Inc, Chicago). *p* < 0.05 was considered significant.

## Results

### Receptor Interacting Protein Kinase 2 Expression Was Upregulated in Hypertrophic Cardiomyocytes or Hearts

**FIGURE 1:**
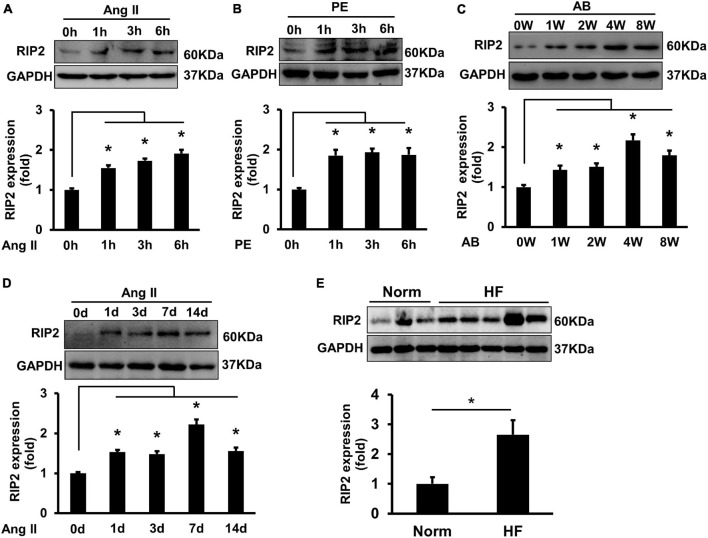
Hypertrophic stimuli enhanced RIP2 expression *in vitro* and *in vivo*. **(A)** Representative Western blot and quantitative results of RIP2 and GAPDH, neonatal rat cardiomyocytes (NRCMs) were treated with angiotensin II (Ang II) for 1, 3, and 6 h, respectively. **(B)** Representative Western blot and quantitative results of RIP2 and GAPDH, NRCMs were treated with phenylephrine (PE) for 1, 3, and 6 h, respectively. **(C)** Representative Western blot and quantitative results of RIP2 and GAPDH in mouse heart after artic banding (AB) surgery at 0, 1, 2, 4, and 8 weeks, respectively (*n* = 6). **(D)** Representative Western blots and quantitative results of RIP2 and GAPDH after Ang II treatment in mouse at 1, 3, 7, and 14 days, respectively. **(E)** Representative Western blots and quantitative results of RIP2 and GAPDH in human hearts (normal heart = 6, failing heart = 8). Protein expression was normalized to GAPDH before quantitative analysis. Data are presented as mean ± SEM, Statistical analysis was performed by *t*-test or one-way ANOVA according to the number of groups. *p < 0.05 versus indicated group.

Angiotensin II (Ang II, 1 μM) or PE (50 μM) treatment could remarkably enhance RIP2 expression in isolated NRCMs at 1, 3, and 6 h, respectively (Figures 1A,B). RIP2 expression was significantly upregulated after 1, 2, 4, and 8 weeks of pressure overload, respectively, compared to sham surgery group (Figure 1C). In addition, RIP2 expression was also significantly upregulated in Ang II-treated mouse hearts at 1, 3, 7, and 14 days, respectively (Figure 1D). We also presented that RIP2 was significantly upregulated in human failure hearts with dilated cardiomyopathy compared to normal donor hearts (Figure 1E). These results strongly indicated that RIP2 was involved in regulating the process of pathological cardiac remodeling and heart failure.

### Receptor Interacting Protein Kinase 2 Overexpression Aggravated Pressure Overload-Induced Mouse Heart Hypertrophy

**FIGURE 2:**
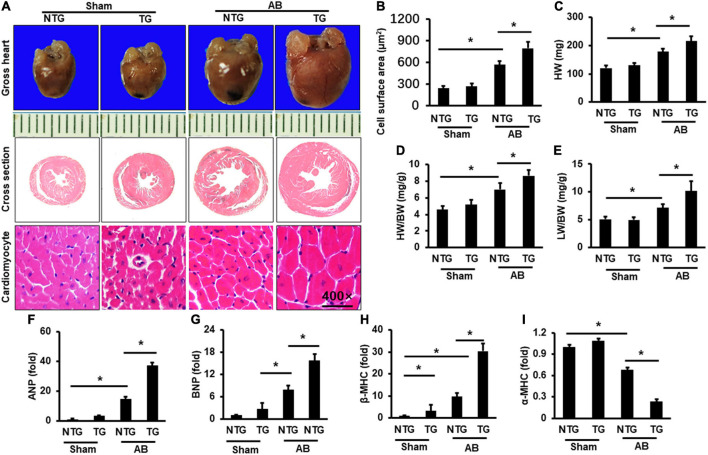
RIP2 overexpression aggravated pressure overload-induced mouse heart hypertrophy. **(A)** Gross heart and HE staining for mouse heart slices (*n* = 6). **(B)** Assessed cardiomyocyte cross-section areas (*n* = 6). **(C)** Calculated mouse heart weight (HW) (*n* = 12). **(D)** The ratios of HW/body weight (HW/BW) (*n* = 12). **(E)** The ratios of lung weight to BW (LW/BW). **(F–I)** Quantitative PCR was performed to examine the mRNA expression of ANP **(F)**, BNP **(G)**, β-MHC **(H)**, and α-MHC **(I)** (*n* = 6). Data are presented as mean ± SEM. Statistical analysis was performed by one-way ANOVA. *p < 0.05 versus indicated group.

RIP2 transgene mice (TG) was constructed as described in Supplementary Figure 1. Western blots were used to test the overexpression of RIP2 in mouse heart (Supplementary Figure 1B). Mice with intermediate expression of RIP2 were selected for the following experiments (Supplementary Figure 1B). Immunofluorescence staining was used to indicate that RIP2 has specific overexpression in cardiomyocytes of TG mice (Supplementary Figure 2). TG mice and their littermate mice (also named non-transgenic mice, NTG) were subjected to AB surgery for 4 weeks to establish mouse model of pressure overload-induced cardiac hypertrophy. As shown in Figure 2, pressure overload markedly induced cardiac hypertrophy evidenced by enlarged cardiomyocyte surface area (CSA) and increased heart weight (HW), heart weight/body weight (HW/BW), and lung weight/body weight (LW/BW) compared to the Sham group (Figures 2A–E); however, RIP2 overexpression further exacerbated cardiac hypertrophy compared with the NTG + AB group evidenced by further increased CSA, HW, HW/BW, and LW/BW (Figures 2A–E). No significant difference in CSA, HW, HW/BW, and LW/BW was detected between the NTG and TG group without AB surgery (Figures 2A–E). Consistently, pressure overload significantly increased the mRNA expression of hypertrophy-associated markers including atrial natriuretic peptide (ANP), brain natriuretic peptide (BNP), and β-myosin heavy chain (β-MHC, a marker of the fetal gene reversion program) but decreased α-myosin heavy chain (α-MHC) expression in the NTG + AB group compared to the Sham group (Figures 2F–I). However, RIP2 overexpression further significantly increased expression of ANP, BNP, and β-MHC but decreased α-MHC expression (Figures 2F–I). Moreover, we also noted that RIP2 expression could increase β-MHC expression in the TG group compared to the NTG group without AB surgery (Figure 2H), but there was no difference in mortality between the NTG and TG group after AB operation for 4 weeks (data not shown).

### Receptor Interacting Protein Kinase 2 Overexpression Exacerbated Aortic Banding-Induced Cardiac Fibrosis

**FIGURE 3:**
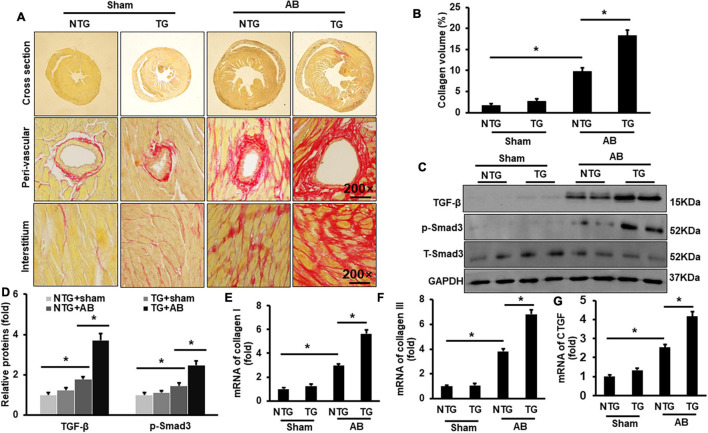
RIP2 overexpression aggravated pressure overload-induced mouse cardiac fibrosis. **(A)** PSR staining for mouse heart slices (*n* = 6). **(B)** Assessed collagen volume in mouse heart (*n* = 6). **(C)** Western blots of TGF-β, p-Smad3, T-Smad3, and GAPDH (*n* = 6). **(D)** Relative quantitative results of TGF-β, p-Smad3, and T-Smad3 (*n* = 6). **(E–G)** Quantitative PCR was performed to examine the mRNA expression of collagen I **(E)**, collagen II **(F)**, and CTGF **(G)** (*n* = 6). Protein or mRNA expression was normalized to GAPDH before quantitative analysis. Data are presented as mean ± SEM. Statistical analysis was performed by one-way ANOVA. *p < 0.05 versus indicated group.

Picrosirius red (PSR) was performed to evaluate cardiac fibrosis. Pressure overload induced significant fibrosis in the AB group compared to the Sham groups (Figures 3A,B). RIP2 overexpression further exacerbated pressure overload-induced cardiac fibrosis (Figures 3A,B). Pressure overload significantly activated the TGF-β/Smad3 signaling pathway in the NTG+AB group compared to the NTG + Sham group (Figures 3C,D), which could be further strengthened in the TG + AB group compared to the NTG + AB group (Figures 3C,D). Consistently, the mRNA expression of fibrosis-associated markers, including collagen I, collagen III, and connective tissue growth factor (CTGF), was significantly upregulated in the NTG + AB group compared to the NTG + Sham group (Figures 3E–G). RIP2 overexpression further significantly boosted expression of these fibrosis-associated markers (Figures 3E–G).

### Receptor Interacting Protein Kinase 2 Overexpression Aggravated Pressure Overload-Induced Cardiac Dysfunction

**FIGURE 4:**
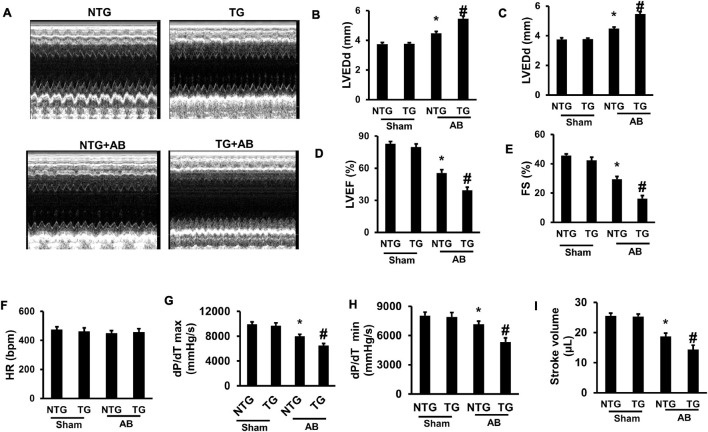
RIP2 overexpression aggravated pressure overload-induced mouse cardiac dysfunction. **(A)** The representative M-mode images of each indicated group. **(B)** Assessed left ventricle end-diastolic dimension (LVEDd) (*n* = 12). **(C)** Assessed left ventricle end-systolic dimension (LVEDs) (*n* = 12). **(D)** Calculated the left ventricle ejection fraction (EF) (*n* = 12). **(E)** Calculated the fraction shortening (FS) (*n* = 12). **(F)** Heart rates (HR) in different groups after inhalation anesthesia with 1.5% isoflurane (*n* = 12). **(G)** Maximal rate of pressure development (dp/dt max) (*n* = 12). **(H)** Minimal rate of pressure development (dp/dt min) (*n* = 12). **(I)** Stroke volume (*n* = 12). Data are presented as mean ± SEM. Statistical analysis was performed by one-way ANOVA. *p < 0.05 versus NTG + Sham group, #*p* < 0.05 versus NTG + AB group.

Echocardiography was performed to evaluate mouse cardiac function. No significant difference was examined between the NTG and TG group without AB surgery (Figures 4A–E). Pressure overload markedly increased left ventricle end-diastolic diameter (LVEDd) and LV- end-systolic diameter (LVEDs) but decreased ejection fraction (EF) and fractional shortening (FS) in the NTG + AB group compared to the NTG + Sham group (Figures 4B–E); RIP2 overexpression further significantly increased LVEDd and LVEDs and decreased EF and FS in the TG + AB group compared to the NTG + AB group (Figures 4B–E). However, there were no differences in heart rate (HR) among the four groups (Figure 4F). In addition, pressure-volume loop analysis showed that pressure overload significantly induced decline of maximal rate of pressure development (dp/dt max), minimum rate of pressure development (dp/dt min), and stroke volume (Figures 4G–I), which could be further exasperated after RIP2 overexpression (Figures 4G–I). Thus, RIP2 overexpression remarkably exaggerated pressure overload-induced mouse heart dysfunction.

### Receptor Interacting Protein Kinase 2 Overexpression Activated the TAK1/JNK1/2/P38 Signaling Pathway

**FIGURE 5:**
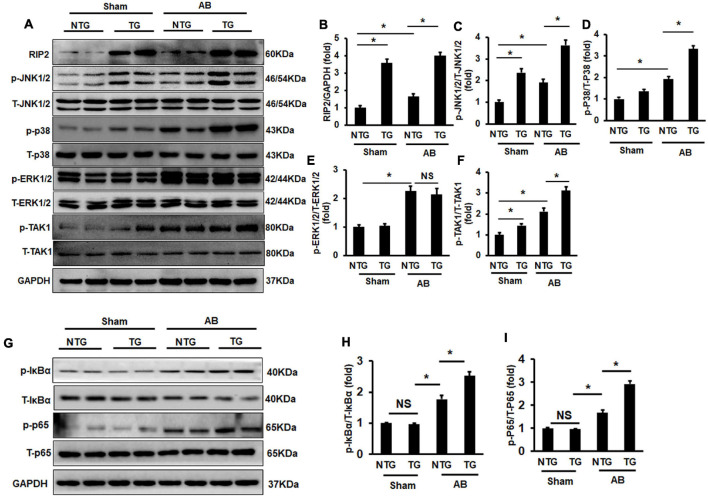
RIP2 overexpression activated TAK1/JNK1/2/P38 signaling. **(A)** Representative Western blots of RIP2, p-TAK1, T-TAK1, p-P38, T-p38, p-JNK1/2, T-JNK1/2, p-ERK1/2, and T-ERK1/2 (*n* = 6). **(B–F)** Quantitative results of RIP2 **(B)**, p-JNK1/2 **(C)**, p-P38 **(D)**, p-ERK1/2 **(E)**, and p-TAK1 **(F)** (*n* = 6). **(G)** Representative Western blots of p-IκBα, IκBα, p-P65, P65, and GAPDH (*n* = 6). **(H,I)** Relative quantitative results of p-IκBα **(H)** and p-P65 **(I)**. Representative protein expression was normalized to GAPDH firstly and then compared to total protein for quantitative analysis. Data are presented as mean ± SEM. Statistical analysis was performed by one-way ANOVA. ns, no significant difference, *p < 0.05 versus indicated group, NS means no significant difference between indicated groups.

RIP2 was suggested to regulate MAPK signaling in previous studies (Humphries et al., 2015), and MAPK signaling participated in regulation of pathological cardiac hypertrophy (Wu et al., 2017). This study firstly evaluated the phosphorylation status in the TAK1/MAPK pathway. Pressure overload induced hyperphosphoryl action of ERK1/2, JNK1/2, p38, and TAK1 in the NTG + AB compared to the NTG + Sham group (Figures 5A–F). RIP2 overexpression further activated phosphorylation of JNK1/2 and p38 in the TG + AB group compared to the NTG + AB group but did not further activate ERK1/2 phosphorylation (Figures 5A–F). Moreover, we could also observe that RIP2 overexpression significantly activated JNK1/2 and TAK1 phosphorylation at baseline (Figures 5A,C,F).

Previous studies have demonstrated that RIP2 could mediate activation of the IKBα/NF-κB/p65 signaling pathway (Humphries et al., 2015; Zhao et al., 2017). In this study, we also demonstrated that pressure overload could cause significant hyperphosphorylation of IκBα and P65 in the NTG + AB group compared to the NTG + Sham group (Figures 5G–I). RIP2 overexpression could further activate the phosphorylation of IκBα and p65 in the TG + AB group compared to the NTG+AB group (Figures 5G–I).

### Treatment of TAK1-Specific Inhibitor Completely Counteracted Receptor Interacting Protein Kinase 2 Overexpression-Mediated Cardiac Remodeling

**FIGURE 6:**
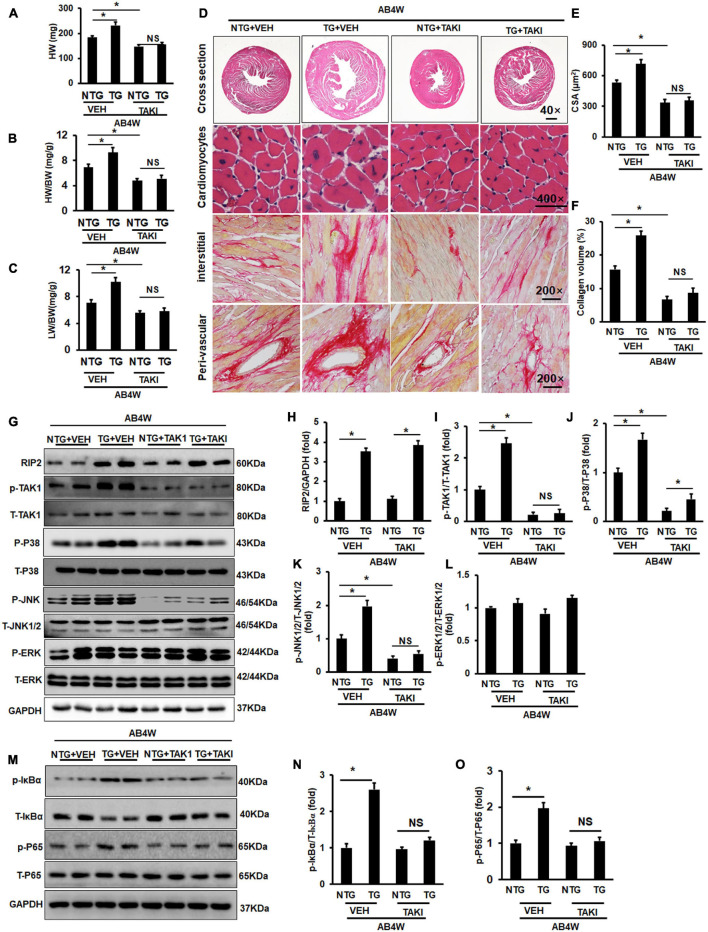
Inhibition of TAK1 thoroughly blocked the pro-hypertrophic effect of RIP2 overexpression. **(A)** Calculated mouse heart weight (HW) (*n* = 12), the “VEH” meant the mice in indicated groups treated with DMSO (the solvent for inhibitor of TAK1), the “TAKI” meant the mice in indicated groups treated with inhibitor of TAK1, and the “AB4W” represented aortic banding operation for 4 weeks. **(B)** Calculated the ratios of HW/body weight (HW/BW) (*n* = 12). **(C)** Calculated the ratios of lung weight/BW (LW/BW) (*n* = 12). **(D)** HE and PSR staining for mouse heart slices (*n* = 6). **(E)** Assessed cardiomyocyte cross-section areas (CSA) (*n* = 6). **(F)** Assessed collagen volume in mouse heart sections (*n* = 6). **(G)** Representative Western blots of indicated proteins (*n* = 6). **(H–L)** Quantitative results of RIP2, p-TAK1, p-P38, p-JNK1/2, and p-ERK1/2, respectively (*n* = 6). **(M)** Representative Western blots of indicated proteins (*n* = 6). **(N,O)** Representative Western blot of relative proteins and the corresponding quantitative results of p-IκBα/T-IκBα and p-P65/T-P65. Protein expression was normalized to GAPDH firstly and then compared to total protein for quantitative analysis. Data are presented as mean ± SEM. Statistical analysis was performed by one-way ANOVA. *p < 0.05 versus indicated group. NS means no significant difference between indicated groups.

Receptor interacting protein kinase 2 overexpression significantly increased HW, HW/BW, and LW/BW in the TG + VEH group (the RIP2 transgene mice treated with DMSO) compared to the NTG + VEH group (the non-transgene littermates treated with DMSO) after AB operation for 4 weeks (Figures 6A–C); however, no significant difference in HW, HW/BW, and LW/BW could be observed between the TG + TAKI (the RIP2 transgene mice treated with inhibitor of TAK1) and the NTG + TAKI group (the non-transgene littermates treated with inhibitor of TAK1) (Figures 6A–C). RIP2 overexpression remarkably exaggerated pressure overload-induced cardiomyocyte hypertrophy and interstitial fibrosis, which also exhibited no significant difference between the TG + TAKI and NTG + TAKI group (Figures 6D–F). Mechanistically, TAKI-specific inhibitor completely inhibited RIP2-mediated hyperphosphorylation of TAK, JNK1/2, and P38 (Figures 6G–L); moreover, TAKI could offset the activation the IκBα and P65 attributed to RIP2 overexpression after AB for 4 weeks (Figures 6M–O).

### Receptor Interacting Protein Kinase 2 Overexpression Exacerbated Cardiomyocyte Hypertrophy and Binding to TAK1 *in vitro*

**FIGURE 7:**
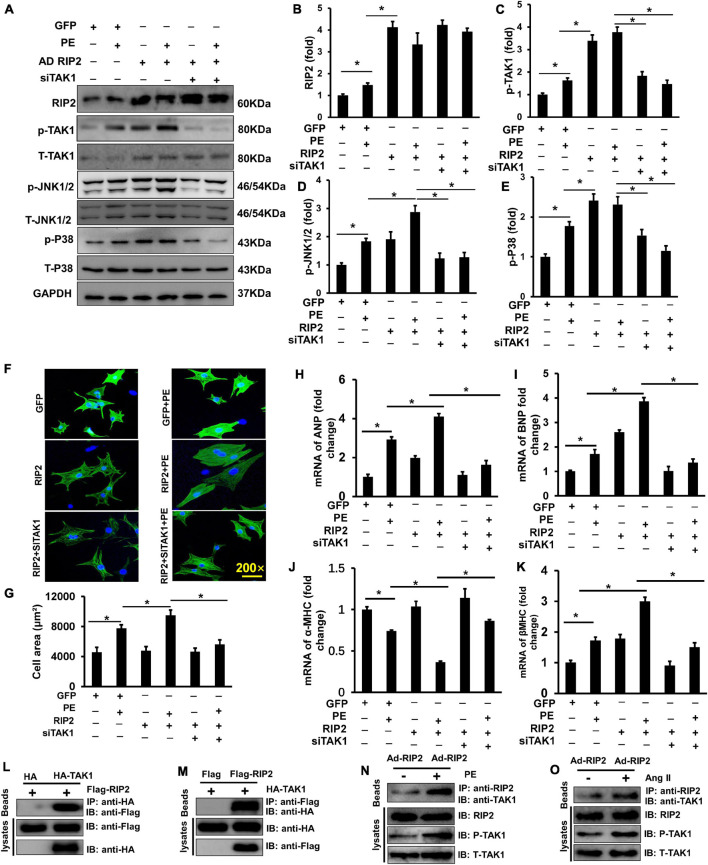
RIP2 overexpression exacerbated cardiomyocyte hypertrophy and binding to TAK1. **(A)** Representative Western blots of RIP2, p-TAK1, T-TAK1, p-JNK1/2, T-JNK1/2, p-P38, and T-p38 after co-transfecting with AdRIP2 or siRNA for TAK1(siTAK1) in PE-induced hypertrophic cellular model. **(B–E)** Quantitative results of RIP2 **(B)**, p-TAK1 **(C)**, p-JNK1/2 **(D)**, and p-P38 **(E)**. **(F)** Immunofluorescence staining of NRCMs with the anti-α-actinin antibody after PE treatment for 48 h. **(G)** Assessed surface area of NRCMs after PE treatment for 48 h. **(H–K)** Quantitative PCR was performed to examine the mRNA expression levels of ANP, BNP, α-MHC, and β-MHC after PE treatment for 48 h. **(L,M)** HEK293T cell was co-transfected with pcDNA-HA-TAK1 or pcDNA-Flag-RIP2. After incubating for 48 h, HEK293T cell was harvested and was lysed. Cellular lysates were used for immunoprecipitation (IP) with antibodies against HA **(L)** or Flag **(M)**. **(N,O)** The PE **(N)** and Ang II **(O)** exacerbate the interaction of RIP2 and TAK1, and activate TAK1. Protein expression was normalized to GAPDH firstly and then compared to total protein for quantitative analysis. mRNA expression was normalized to GAPDH for relative quantitative analysis. Data are presented as mean ± SEM. Statistical analysis was performed by one-way ANOVA. *p < 0.05 versus indicated group.

To further clarify whether the hyperphosphorylation of TAK1/JNK1/2/P38 signaling was essential for RIP2-mediated cardiomyocyte hypertrophy, NRCMs was isolated and transfected with adenovirus for RIP2 overexpression (Ad-RIP2) or specific small interfering RNA for TAK1 silence (siTAK1), as shown in Figure 7. Ad-RIP2-mediated RIP2 overexpression significantly activated phosphorylation of TAK1, JNK1/2, and P38 compared to the PE + GFP group (Figures 7A–E); however, siTAK1-mediated TAK1 silence completely blunted RIP2 overexpression-induced JNK1/2 and P38 phosphorylation compared to the PE + Ad-RIP2 group (Figures 7A,D,E). We also observed that RIP2 overexpression further enlarged PE-mediated NRCMs hypertrophy in the PE + Ad-RIP2 group compared to PE treatment group, which could be prevented by TAK1 silence *via* co-transfecting with siTAK1 (Figure 7F). Consistently, RIP2 overexpression notably exaggerated PE-induced expression of fetal genes, including ANP, BNP, and β-MHC in the PE + Ad-RIP2 group compared to the PE group (Figures 7H,I,K), and further exaggerated α-MHC downregulation in the PE + Ad-RIP2 group compared to the PE + GFP group (Figure 7J). Co-transfection of siTAK1 completely inhibited RIP2-mediated upregulation of ANP, BNP, and β-MHC, and downregulation of α-MHC (Figures 7H–K).

By transfecting HEK293T cells with HA-tagged TAK1 and Flag-tagged RIP2, we found that RIP2 could combine with TAK1 (Figures 7L,M). Through immunoprecipitation experiments in NRCM, we also demonstrated that PE or Ang II treatment could promote interaction between TAK1 and RIP2 as well as activate TAK1 phosphorylation (Figures 7N,O). These investigations confirmed that RIP2 overexpression contributed to cardiomyocyte hypertrophy by mediated phosphorylation of JNK1/2 and p38 *via* directly recruited and activated TAK1.

### Receptor Interacting Protein Kinase 2 Silence Could Inhibit Phenylephrine-Induced Neonatal Rat Cardiomyocytes Hypertrophy *in vitro*

**FIGURE 8:**
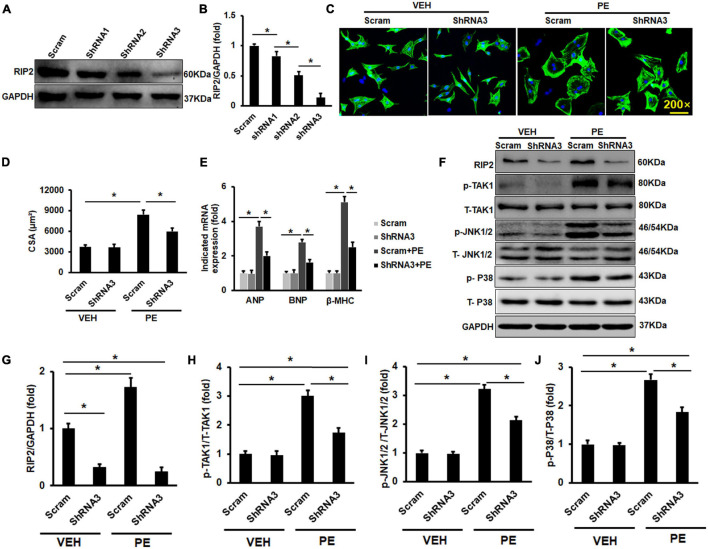
RIP2 silence could inhibit PE-induced NRCMs hypertrophy *in vitro*. **(A)** The Western blot results of RIP2 expression treated with different RI2-shRNA. **(B)** Quantitative results of RIP2 protein level. **(C)** Representative images for NRCMs with indicated treatment. **(D)** The average cross-section areas (CSA) in indicated groups. **(E)** The mRNA levels of hypertrophic markers in indicated groups. **(F)** The Western blot results of p-TAK1, p-JNK1/2, and p-P38 in NRCMs challenged with VEH or PE after infected with indicated adenovirus. **(G–J)** The protein quantitative analysis of RIP2 **(G)**, p-TAK1 **(H)**, p-JNK1/2 **(I)**, and p-P38 **(J)**. Protein expression was normalized to GAPDH firstly and then compared to total protein for quantitative analysis. mRNA expression was normalized to GAPDH for relative quantitative analysis. Data are presented as mean ± SEM. Statistical analysis was performed by one-way ANOVA. *p < 0.05 versus indicated group.

Because this study has presented that pro-hypertrophic stimuli could enhance RIP2 overexpression, we next investigate whether RIP2 knockdown could inhibit PE-induced NRCM hypertrophy. Adenovirus-mediated shRNA expression was used to knock down RIP2 expression *in vitro* (Figures 8A,B). PE treatment could promote NRCM hypertrophy significantly; however, RIP2 knockdown could remarkably prevent PE-induced NRCM hypertrophy (Figures 8C,D). Consistently, the hypertrophy-associated markers including ANP, BNP, and β-MHC were significantly upregulated in the PE group compared to the VEH group (Figure 8E). These upregulated markers were markedly inhibited in the PE + shRNA group compared to the PE + Scram group (Figure 8E). Mechanistically, PE treatment significantly induced upregulation of RIP2 and hyperphosphorylation of TAK1, JNK1/2, and P38 (Figures 8F–J); however, RIP2 knockdown significantly inhibited PE-induced hyperphosphorylation of TAK1, JNK1/2, and P38 (Figures 8F–J).

### Receptor Interacting Protein Kinase 2 Overexpression Resulted in Spontaneous Cardiac Hypertrophy and Fibrosis

**FIGURE 9:**
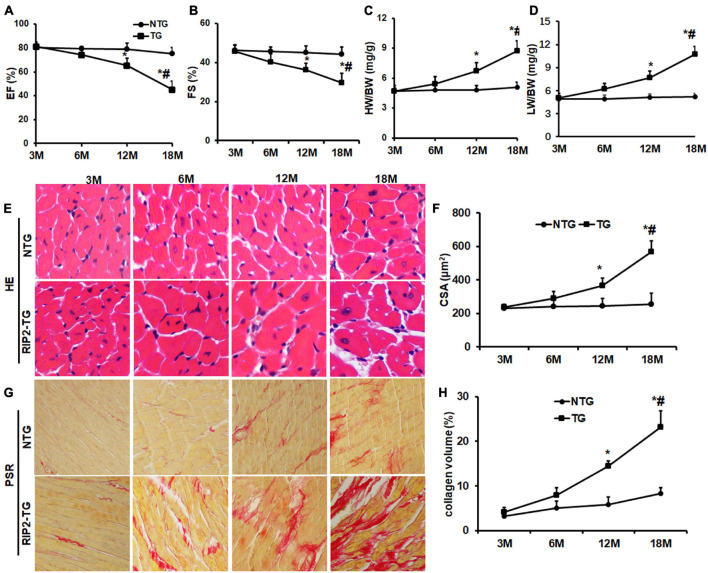
RIP2 overexpression resulted in spontaneously cardiac dysfunction, hypertrophy and fibrosis. **(A)** The left ventricle ejection fraction (EF) in NTG and TG mice at the indicated age (*n* = 10). **(B)** The fractional shortening (FS) in NTG and TG mice at the indicated age (*n* = 10). **(C)** The ratios of HW/BW at the indicated age (*n* = 10). **(D)** The ratios of LW/BW at the indicated age (*n* = 10). **(E)** The representative HE staining images at the indicated age (*n* = 6). **(F)** The cardiomyocyte areas in NTG and TG mice at different ages (*n* = 6). **(G)** The representative pictures of PSR staining at the indicated age (*n* = 6). **(H)** Quantitative collagen deposition in each group at the indicated age. Data are presented as mean ± SEM. Statistical analysis was performed by *t*-test. *p < 0.05 versus NTG group at same age. ^#^*p* < 0.05 means the corresponding difference value of indicators between the NTG group and TG group at the age of 18 months compared to 12 months.

There were no significant differences in EF and FS between the NTG group and TG group at the age of 3 and 6 months. However, the TG mice showed a significant reduction of EF and FS compared with NTG mice at the age of 12 months, which were further decreased significantly in the 18-month-old TG group compared to the 12-month-old group (Figures 9A,B). Consistently, mice in the 12-month-old TG group had a significant higher HW/BW and LW/BW compared to the 12-month-old NTG group, which was further increased in the 18-month-old TG group compared to the 12-month-old TG group (Figures 9C,D). HE and PSR staining exhibited that RIP2 overexpression caused a significant cardiomyocyte hypertrophy and myocardial fibrosis spontaneously in the 12- and 18-month-old TG groups compared to the NTG mice of the same age group (Figures 9E–H). The deterioration of RIP2 overexpression was more significant in the TG group at age of 18 months compared to 12 months (Figures 9E–H). Taken together, RIP2 overexpression further aggravated spontaneous cardiac hypertrophy and fibrosis as aging.

### Receptor Interacting Protein Kinase 2 Knockout Attenuated Pressure Overload-Induced Cardiac Remodeling *in vivo*

**FIGURE 10:**
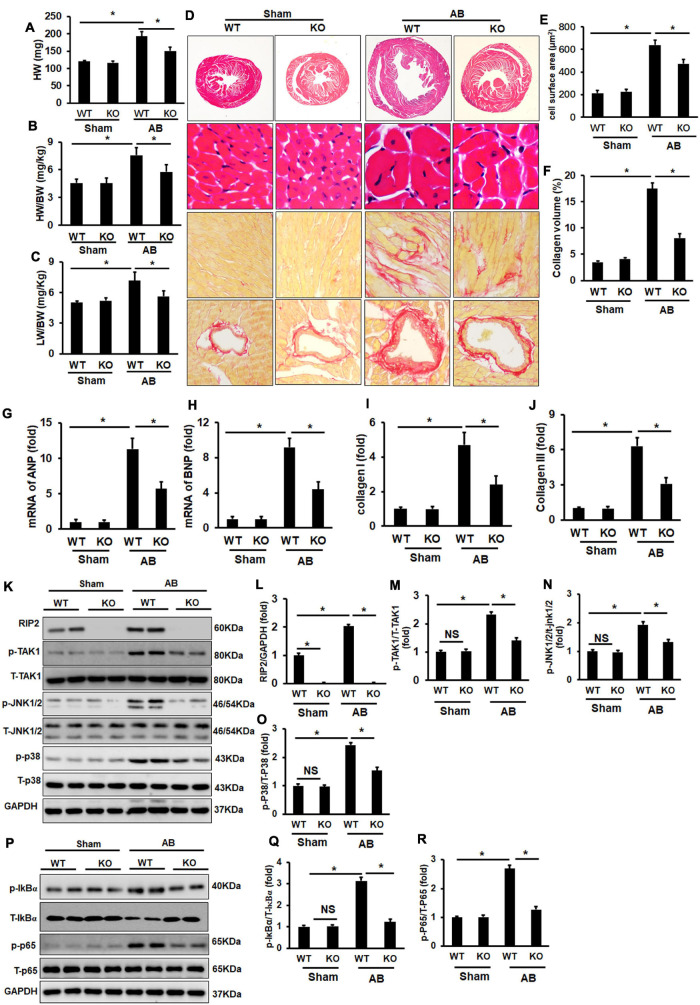
RIP2 knockout attenuated pressure overload-induced cardiac remodeling. **(A–C)** The heart weight (HW) of wild-type mice (WT) and RIP2 global knockout (KO) mice subjected with AB or sham operation **(A)**; calculated the ratios of HW/body weight (HW/BW) **(B)**; calculated the ratios of lung weight/BW (LW/BW) **(C)**. **(D)** HE and PSR staining for mouse heart slices (*n* = 6). **(E)** Assessed cardiomyocyte cross-section areas (CSA) (*n* = 6). **(F)** Assessed collagen volume in mouse heart sections (*n* = 6). **(G–J)** Relative mRNA levels of ANP **(G)**, BNP **(H)**, Collagen I **(I)**, and Collagen III **(J)**. **(K)** Representative Western blots of RIP2, p-TAK1, T-TAK1, p-P38, T-p38, p-JNK1/2, T-JNK1/2, and GAPD (*n* = 6). **(L–O)** Quantitative results of RIP2 **(L)**, p-TAK1 **(M)**, p-JNK1/2 **(N)**, and p-P38 **(O)** (*n* = 6). **(P)** Representative Western blots of p-IκBα, p-P65, T-P65, and GAPDH. **(Q)** The fold change of p-IκBα in four groups. **(R)** The fold change of p-P65 in the indicated groups. Protein expression was normalized to GAPDH firstly and then compared to total protein for quantitative analysis. The mRNA level was normalized to GAPDH and then for quantitative analysis. Data are presented as mean ± SEM. Statistical analysis was performed by one-way ANOVA. *p < 0.05 versus the indicated group.

Pressure overload significantly induced cardiac hypertrophy evidenced by increased HW, HW/BW, LW/BW, CSA, and collagen deposition, all of which could be significantly attenuated by RIP2 knockout (Figures 10A–F). Consistently, pressure overload promoted overexpression of pro-hypertrophy and fibrosis-associated genes including ANP, BNP, collagen I, and collagen III, all of which could be significantly downregulated after RIP2 knockout (Figures 10G–J). Mechanistically, RIP2 knockout significantly inhibited hyperphosphorylation of TAK1, JNK1/2, P38, P65, and IκBα (Figures 10K–R).

## Discussion

This study revealed that RIP2 was upregulated in failing human hearts with dilated cardiomyopathy and mouse hearts or NRCMs challenged with pro-hypertrophy stimuli. RIP2 overexpression exacerbated pathological cardiac hypertrophy and caused spontaneous cardiac hypertrophy, which had not been reported previously. RIP2 knockout *in vivo* or knockdown *in vitro* could remarkably attenuate pathological-associated remodeling. According to our *in vivo* and *in vitro* remodeling models, we clearly demonstrated that RIP2 overexpression activated TAK1/JNK1/2/P38 and IκBα/P65 signaling. Moreover, immunoprecipitation indicated that the RIP2–TAK1 interaction might be essential for RIP2-mediated malignant activation of TAK1/JNK1/2/P38 and IκBα/P65 signaling.

This study demonstrated that RIP2 overexpression could significantly promote p38 and JNK1/2 phosphorylation activation. Many studies have exhibited that the activation of MAPK (p38, JNK1/2, and ERK1/2) signaling during pathological stress accelerated the process and development of cardiac hypertrophy and transformation to heart failure (Rose et al., 2010; Yokota and Wang, 2016; Gallo et al., 2019). MAPKs are key signaling transduction nodes that receive many upstream signaling and transmit tens of signal pathways to downstream (Rose et al., 2010). MAPK signaling regulated many biological events including proliferation, differentiation, metabolism, survival, and apoptosis (Rose et al., 2010). Direction inhibition of MAPKs might disrupt some normal physiological functions; thus, investigators have been trying their best to seek upstream regulating mechanisms of MAPK activation with the hope of finding out new therapeutic ways to treat malignant cardiac remodeling or heart failure without disrupting physiological functions. This study clearly demonstrated that RIP2 was an upstream regulating kinase of MAPK, and inhibiting RIP2 upregulation might be a potential strategy for preventing malignant cardiomyocyte hypertrophy.

Li et al. (2015) showed that RIP2 overexpression could aggravate myocardial infarction-associated cardiac remodeling by boosting p38 hyperphosphorylation. RIP2 could also cause myocardial p38 dual phosphorylation in endotoxin component MDP-treated mouse heart (Jacquet et al., 2008). However, in another article of exposure to MDP before ischemia–reperfusion injury, RIP2-dependent activation of TAK1/JNK1/2 MAPK signaling markedly reduced infarction mouse heart size (Sicard et al., 2009). In triple-negative breast cancer cell, RIP2 overexpression promoted cell migration and invasion *via* activating JNK1/2 and NF-κB signaling pathways (Singel et al., 2014). These studies showed that RIP2 seemed to regulate p38/MAPK or JNK1/2 MAPK depending on different cell or disease circumstances, respectively. We firstly demonstrated that TAK1 inhibition could completely prevent RIP2 overexpression-induced malignant mouse heart hypertrophy.

TAK1 is a key molecular node and locates at upstream of MAPK signaling (Ji et al., 2016; Li et al., 2016). TAK1 overactivation under different disease conditions could significantly exacerbate malignant cardiac hypertrophy (Ji et al., 2016; Li et al., 2016); however, TAK1-specific ablation in mouse heart caused spontaneous cardiac remodeling and heart failure (Li et al., 2014). Because TAK1 plays important roles in regulating many physiological activities, including cell apoptosis (Li et al., 2014), innate and adaptive immune (Sato et al., 2005), development (Jadrich et al., 2006), and cardiomyocyte differentiation (Monzen et al., 2001), it is necessary to find out a way to maintain TAK1 phosphorylation at its physiological level neither over-activation nor excessive inhibition in treating cardiac remodeling. This study indicated that RIP2 located at the upstream of TAK1, inhibiting RIP2 upregulation, might avoid disturbance of normal physiological activities of TAK1.

In this manuscript, we firstly exhibited that RIP2 overexpression in cardiomyocytes aggravated pressure overload-induced cardiac remodeling and led to spontaneous cardiac remodeling. In addition, our research also exhibited that global deletion of RIP2 could prevent pressure overload-induced cardiac hypertrophy. Consistently, Zhao et al. (2017) reported that RIP2 knockout could mitigate AB-induced cardiac hypertrophy, fibrosis, and inflammation *via* reducing TLR4/MyD88/NF-κB, MAPKs, and TGF-β/Smad signaling. We further clarified in this study that RIP2 mediated MAPK (JNK1/2 and p38) and IκBα/p65 activation *via* binding and phosphorylating TAK1. Recently, Lin et al. (2020) demonstrated that RIP2 upregulation could promote mitochondrial antiviral signaling protein (MAVS) expression, exaggerating pressure overload-induced pathological remodeling. Our data in this study and these published findings suggested that RIP2 inhibition might be a potential strategy for treating malignant cardiac remodeling.

Another problem was how RIP2 was upregulated and activated in cardiomyocytes challenged with pro-hypertrophic stimuli. MDP-mediated NOD2 oligomerization contributes to the classical activation of RIP2 activity resulting in activating IκBα/NF-κB/p65 and MAPKs/AP1 signaling (Humphries et al., 2015). Obviously, RIP2 activation in this manuscript was not from MDP*-*mediated NOD2 oligomerization. Except for NOD2-mediated RIP2 activity regulation, several E3 ubiquitin ligases, such as Pellino3, ITCH, TRAF6, cIAP, and XIAP, have been suggested to catalyze RIP2 ubiquitination. ITCH-mediated RIP2 ubiquitination has been demonstrated to inhibit NF-κB signaling. From these investigations, we know that the upstream regulation pathway of RIP2 is complex, and some other investigations are necessary to clarify the upstream regulating mechanism of RIP2 in the process and development of malignant cardiac hypertrophy in succeeding studies.

## Conclusion

This study clearly presented that RIP2 was upregulated and was a key molecular switch for the development and progress of pathological cardiac hypertrophy. RIP2 overexpression could lead to spontaneous cardiac dysfunction and remodeling even without challenging pressure overload, which meant that RIP2 was a key regulator for cardiac remodeling and was a powerful target for treating cardiac remodeling. Mechanically, upregulated RIP2 after pathological stimuli could contribute to RIP2–TAK1 interactions, resulting in TAK1 phosphorylation and activation, which further induced phosphorylation and activation of MAPKs (JNK1/2 and p38) and IκBα/P65, resulting in malignant cardiac hypertrophy. Thus, inhibition of RIP2 upregulation or its interaction with TAK1 might be a potential strategy for preventing pathological cardiac hypertrophy.

## Data Availability Statement

The original contributions presented in the study are included in the article/Supplementary Material, further inquiries can be directed to the corresponding author/s.

## Ethics Statement

The studies involving human participants were reviewed and approved by the Ethics Committee of Renmin Hospital of Wuhan University. The patients/participants provided their written informed consent to participate in this study. The animal study was reviewed and approved by Animal Care and Use Committee of Renmin Hospital of Wuhan University.

## Author Contributions

J-JY and NZ finished most of the work in this study (including WB, qPCR, HE, and PSR staining). JN and HF were responsible for data collection and analysis. W-JL, Z-YZ, and S-QM completed the extraction of neonatal rat cardiomyocytes. H-MW was responsible for animal operation. H-HL and Q-ZT designed and conducted this study. All authors contributed to the article and approved the submitted version.

## Conflict of Interest

The authors declare that the research was conducted in the absence of any commercial or financial relationships that could be construed as a potential conflict of interest.

## Publisher’s Note

All claims expressed in this article are solely those of the authors and do not necessarily represent those of their affiliated organizations, or those of the publisher, the editors and the reviewers. Any product that may be evaluated in this article, or claim that may be made by its manufacturer, is not guaranteed or endorsed by the publisher.

## Supplementary Material

Supplementary Figure 1 **(A)** The schematic diagram of construction of specifically overexpressing RIP2 in cardiomyocytes. **(B)** The western-blot result of RIP2 protein and fold changes of RIP2 in different transgenic lines. *^∗^P* < 0.05 versus indicated group.

Supplementary Figure 2 Immunofluorescence staining detected RIP2 expression in mouse heart, red represented α-actin, green represented rip2, blue represent nucleus. Negative: without addition of RIP2 and α-actin antibody in the staining process, WT, wild type; KO, knockout; TG, Transgene.
