# Supplementary figures and images for: Cardiomyocyte-Specific RIP2 Overexpression Exacerbated Pathologic Remodeling and Contributed to Spontaneous Cardiac Hypertrophy
Source: Front Cell Dev Biol. 2021 Oct 18;9:688238. doi: 10.3389/fcell.2021.688238 (PMC8559979; doi:10.3389/fcell.2021.688238)

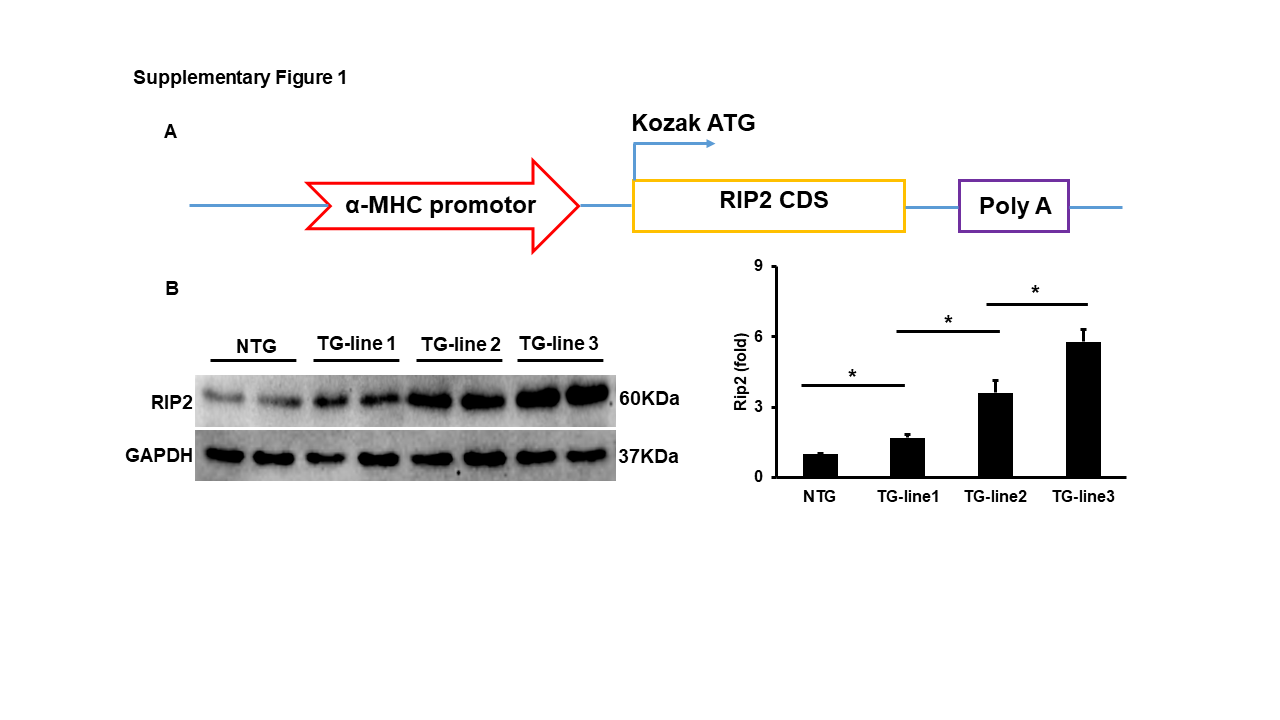

Supplement: Supplementary Figure 1 — (A) The schematic diagram of construction of specifically overexpressing RIP2 in cardiomyocytes. (B) The western-blot result of RIP2 protein and fold changes of RIP2 in different transgenic lines. ∗P < 0.05 versus indicated group. [file Image_1.TIF]

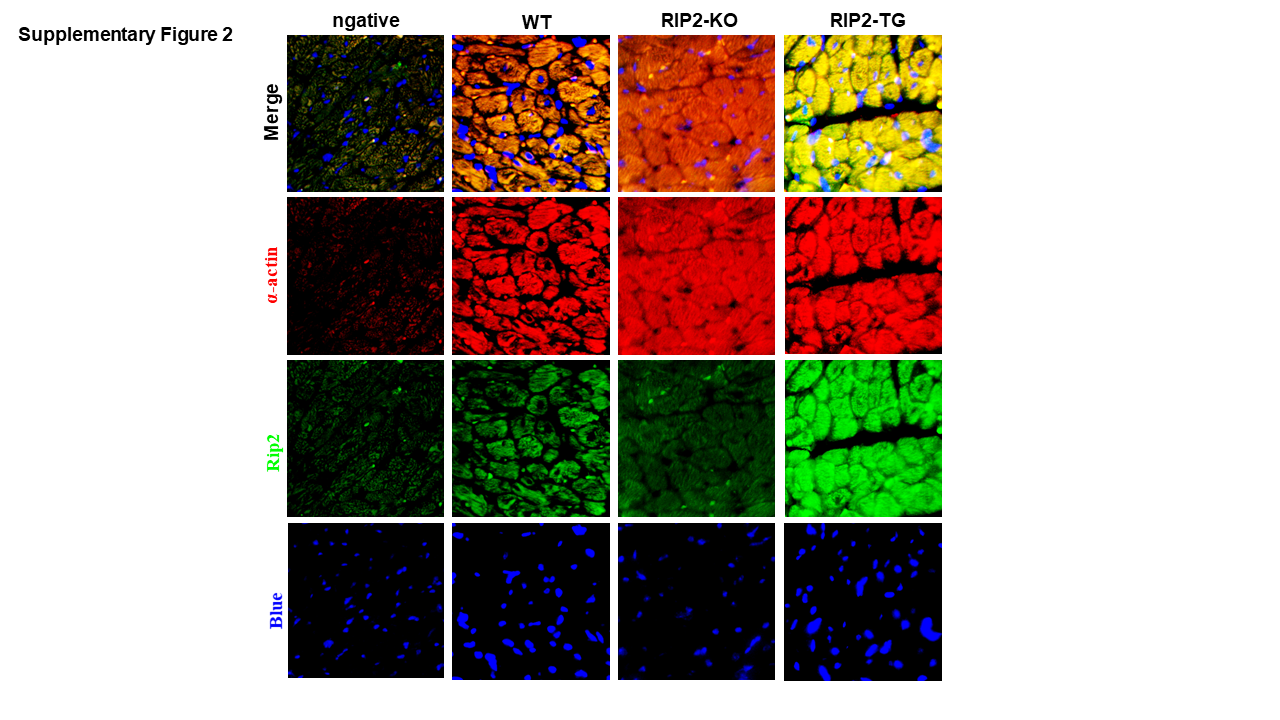

Supplement: Supplementary Figure 2 — Immunofluorescence staining detected RIP2 expression in mouse heart, red represented α-actin, green represented rip2, blue represent nucleus. Negative: without addition of RIP2 and α-actin antibody in the staining process, WT, wild type; KO, knockout; TG, Transgene. [file Image_2.TIF]
